# Beyond cultural competence: a qualitative analysis of psychologists’ experiences with Muslim clients in South Africa

**DOI:** 10.3389/fpsyg.2026.1884512

**Published:** 2026-09-17

**Authors:** Mariam Salie, Ashraf Kagee

**Affiliations:** Department of Psychology, Stellenbosch University, Stellenbosch, South Africa

**Keywords:** cultural humility, explanatory models, mental health, Muslims, psychology, South Africa

## Abstract

**Introduction:**

Muslim mental health is an emerging field of inquiry in the global South. Understanding how mental health conditions are interpreted within diverse Muslim communities is important for culturally responsive psychological care.

**Methods:**

This qualitative study explored psychologists’ experiences of providing services to Muslim clients in the Western Cape, South Africa, and explored psychologists’ accounts of explanatory models of mental health conditions encountered in therapeutic practice. Semi-structured interviews were conducted with 15 clinical and counseling psychologists practicing in the Western Cape. Data were analyzed using Reflexive Thematic Analysis.

**Results:**

Three themes were constructed. First, psychologists emphasized the importance of understanding Muslim clients within their broader sociocultural and historical context, particularly the enduring influence of Apartheid, migration histories, intergenerational trauma, and institutional mistrust. Second, psychologists reported encountering diverse explanatory models among Muslim clients, including interpretations of distress in relation to religiosity, supernatural influences, stigma, personal weakness, and biomedical understandings. These explanatory models were described as shaping emotions such as fear, guilt, and shame, as well as pathways to help-seeking and engagement with treatment. Third, psychologists highlighted the importance of culturally responsive practice, including reflexive engagement with clients’ religious worldviews, ongoing self-education, culturally informed supervision, and ethical collaboration with Muslim religious leaders.

**Discussion:**

The findings suggest that explanatory models are not solely individual beliefs about MHC but are embedded within broader religious, familial, historical, and sociopolitical contexts. By exploring psychologists’ accounts of therapeutic encounters with Muslim clients in the Western Cape, this study extends explanatory-model scholarship by highlighting how collective histories and relationships with systems of care shape understandings of mental health and help-seeking.

## Introduction

Arthur Kleinman, a psychiatrist and medical anthropologist, introduced the concept of explanatory models (EMs) to underscore the subjective and culturally shaped ways patients understand their illnesses, including their beliefs about causes, course, and treatment (Kleinman, 1978, 1980). He argued that biomedicine often overlooked lived experiences and failed to recognize illness as culturally diverse and personally embedded (Kleinman, 1978, 1980, 1983). His work emphasized patient agency, the importance of engaging with the experiences of patients and their families (Kleinman, 1988), and the moral significance of caregiving as a reciprocal practice responsive to the patient’s lifeworld (Kleinman, 2013).

The EM framework has since been widely applied across global health and mental health contexts, demonstrating its value in revealing how patients interpret their conditions and how these interpretations shape help-seeking behavior, treatment adherence, and engagement with care (Aidoo and Harpham, 2001; Clark et al., 2012; Laws, 2016; Lynch and Medin, 2006; Weiss et al., 2006). Research shows that patients’ causal explanations, shaped by personal, social, and cultural factors, strongly influence their illness responses (Apers et al., 2023; Bassett, 2011; Arabiat et al., 2021). Understanding EMs helps identify barriers such as limited mental health literacy or reliance on informal supports (Habte et al., 2016; Lilhare et al., 2020), enabling clinicians to design patient-centered and culturally grounded interventions that improve adherence and outcomes (Apers et al., 2023; Bokhour et al., 2012; Arabiat et al., 2021).

Different cultural and religious beliefs and practices may influence how health conditions are conceptualized (Ibrahim and Whitley, 2020), particularly in ethnic and minority groups (Amusala, 2025), like Muslims. Cultural and religious causal attributions of mental health conditions (MHC) within Muslim communities often include spiritual causes, such as lack of religiosity and supernatural^1^ explanations (Alharbi et al., 2023; Alqasir, 2024; Tanhan and Young, 2022; Ibrahim and Mojab, 2023), and these attributions influence the uptake of mental healthcare, which can in turn influence mental health (MH) outcomes (Amusala, 2025).

For Muslims, the second largest (Hackett et al., 2025; Mitha, 2020) and fastest growing religious group in the world (Hackett and Lipka, 2018), *Qur’an* and prophetic tradition are guides to navigating life and religion typically influences decision making (Badran et al., 2025), including decisions related to mental health. In South Africa, Muslims form an integral part of the multicultural society, with a complex socio-political history (Vahed, 2021). Muslims constitute approximately 2% of South Africa’s population, with notable concentrations in the Western Cape (5.2%) (Statistics South Africa, 2023). Their presence reflects three historical migration streams: (1) enslaved persons, political exiles, and laborers from Southeast Asia and East Africa, (Baderoon, 2014; Günther, 2018); (2) Indian merchants and traders (Günther, 2018); and (3) post-Apartheid migrants from African countries with significant Muslim populations (Günther, 2018; Vawda, 2017). In addition, the Muslim population includes people from indigenous groups, like Zulu, Xhosa and Nguni, which has seen a steady rise since the 1950’s (Ngubane and Sukdaven, 2024; Nkuna, 2016; Sitoto, 2018; Vahed, 2021). While these groups share religious affiliation, their cultural and historical experiences differ markedly. Most Muslims also form part of the previously marginalized racial minorities of South Africa (Vahed, 2021).

Several South African studies have explored Muslim perceptions of MHC, primarily among individuals with prior mental health knowledge. Findings consistently show that Muslims draw on both biomedical and religious explanatory frameworks. Volunteer counselors, psychologists, and general practitioners acknowledged biomedical understandings while also attributing MHC to spiritual factors such as spirit possession, black magic, divine testing, and other supernatural influences, and recommended collaboration with religious leaders in treatment (Laher and Khan, 2011; Laher and Ismail, 2012; Mohamed-Kaloo and Laher, 2014). Collectively, these dated, largely Johannesburg-based studies suggest that Muslim explanatory models of MHC incorporate both biomedical and religious understandings. However, comprehensive exploration of cultural and religious taxonomies remains lacking Sub-Saharan Africa, despite evidence that religion significantly influences wellbeing through practices such as prayer and fasting (Baloch et al., 2014; Newman and Graham, 2018).

This study is framed in the work of Kleinman, who defines explanatory models (EM) as—“notions about an episode of sickness and its treatment that are employed by all those involved in the clinical process” (Kleinman, 1980, p. 105). The study is further framed by a social constructionist approach, acknowledging that meaning is created collectively through shared culture and experiences (Rachmad, 2025). We were guided by the following research questions: What are psychologists’ experiences providing psychotherapeutic services to Muslim clients? How do psychologists describe and engage with explanatory models of MHC as expressed by Muslim clients?

## Methods

### Study overview

This study formed part of a larger PhD study exploring the explanatory models of MHC among Muslims in the Western Cape, South Africa, from a multi-stakeholder perspective. The Western Cape was selected because it contains the largest concentration of Muslims in South Africa and has a distinctive sociocultural history shaped by slavery, migration, Apartheid, and post-Apartheid demographic change. These historical and cultural influences make it a particularly relevant context for exploring how explanatory models of mental health are understood and negotiated in psychological practice. Stakeholders included the general Muslim public, Muslims who have been treated for MHC, Islamic scholars from the community and the current study, psychologists who have treated Muslim clients. This paper does not claim to represent Muslim explanatory models directly. Rather, it examines how psychologists construct, encounter, negotiate and respond to explanatory models expressed by Muslim clients in therapeutic practice. The university’s research ethics committee for social, behavioral and education research (REC: SBE) approved the study (Project ID 23353). This qualitative, exploratory study was informed by the principles of reflexive thematic analysis (RTA) (Braun and Clarke, 2012).

### Participants and data collection

Participants included 15 multi-faith clinical and counseling psychologists working in private and public healthcare in the Western Cape, South Africa. Braun and Clarke (2021) argue that data saturation contrasts the epistemological assumptions of RTA, therefore this study aimed for data adequacy when sampling participants. Data adequacy was evaluated based on the specificity of the participant group. Thus purposive sampling was utilized to ensure that participants were knowledgeable on the topic, with sufficient clinical experience with Muslim clients. A recruitment poster was shared with local and national professional psychology networks and interested participants contacted the first author. All of the 15 participants who made contact participated in the study and were from local networks. Interviews were conducted both online, using Microsoft Teams, and in-person at participants’ workplace, depending on their preference. Psychologists who were independent practitioners^2^, able to converse in English or Afrikaans, and had experience with Muslim clients were included in the study. Although religion was not a prerequisite, 12 of the 15 participants were Muslim and only three participants were male. Participants had a mean age of 41, and a mean experience of 9 years. At the time of data collection (Mar’22–Aug’23), five participants worked in both public and private healthcare, one worked in private healthcare and higher education, and the remaining nine were full-time in private healthcare.

All participants completed a semi-structured interview, developed from the objectives of the study. Interviews included questions about their experiences of treating Muslim patients, knowledge about Muslims’ understanding of MHC and whether this has changed after working with Muslim patients, whether their knowledge or lack of knowledge about Muslims’ explanatory models implicated the treatment process, whether they had to make any adaptations to the treatment process, and lastly, whether their Muslim patients ever referred to the following regarding MHC—Jinn (spirits), shayateen (devils), marrid (demons), bhut (evil spirits). Questions regarding supernatural entities were introduced toward the latter part of the interview after broader questions regarding explanatory models and treatment experiences. All participants received the same questions and while some prompts may have differed depending on the interview responses, prompts remained aligned with objectives. Although these prompts may have increased discussion of supernatural explanations, participants frequently referred to such explanations throughout interviews independent of direct questioning. Interviews lasted between 40–70 min. The extensive clinical experience of participants in a variety of contexts and over an extensive period of time, the focused scope of the research questions along with the detailed and lengthy interviews, provided sufficient depth and diversity to support the analytical construction of the final themes.

This study was informed by a social constructionist epistemology, which assumes that understandings of mental health are socially, culturally, and historically situated rather than objectively discoverable. From this perspective, participants’ accounts were understood as constructions of their experiences of working with Muslim clients, rather than direct representations of an objective reality. Consistent with Reflexive Thematic Analysis (Braun and Clarke, 2021), themes were viewed as interpretative constructions generated through the interaction between the researchers, participants, and data.

### Data analysis

In-person interviews were audio recorded and fully transcribed. Online interviews were video recorded and transcribed automatically on the Microsoft teams video conferencing platform and checked for accuracy. Data were coded through a hybrid inductive-deductive approach, using Atlas ti.V23, a computer assisted qualitative data analysis software. Following Braun and Clarke’s reflexive thematic analysis approach, the first author immersed themselves in the dataset through repeated engagement with interview recordings and transcripts, generating initial codes and analytic reflections. Coding focused on identifying patterns of meaning relevant to psychologists’ experiences of providing psychotherapy to Muslim clients and their accounts of explanatory models. Theme development involved an iterative and interpretative process that moved beyond clustering similar codes. The authors actively examined relationships across codes, considered underlying assumptions and meanings, and developed themes that captured broader patterns across the dataset. Throughout analysis, themes were refined through reflexive engagement with the data, discussions with the supervisor, and ongoing consideration of the study’s theoretical framework and research questions. Lastly, the process was documented, and the analysis is integrated with existing literature to contextualize findings in this paper (Braun and Clarke, 2012; Joffe, 2012).

### Positionality

The first author’s positionality is shaped by both their professional role and lived experiences. This position afforded them an insider perspective, which supported rapport building, trust, and open engagement during data collection. While this facilitated the research process, the first author remains mindful of the assumptions, blind spots, and risk of over-identification that may have emerged through their shared identities with participants, as a Muslim, clinical psychologist working with Muslim clients. They are also aware of the inherent power in the dual role of researcher-clinician and how this may have influenced disclosures and interpretation. Reflexive journaling, regular consultations with the study supervisor, and multiple discussions with colleagues were utilized to ensure continuous interrogation of assumptions and to maintain reflexivity throughout the study. The authors acknowledge that their own knowledge, experiences and assumptions influenced how meanings were interpreted and themes constructed. Rather than seeking objectivity, reflexive practices were employed to promote transparency and rigor in the analytic process. Consistent with RTA, the researchers did not seek to minimize their influence on the analytic process. Rather, their disciplinary knowledge, professional identities, familiarity with Muslim communities, and theoretical commitments informed the questions asked of the data, the meanings foregrounded during analysis, and the construction of themes. Reflexivity was therefore understood as an ongoing practice of critically engaging with how these perspectives shaped interpretation.

## Results

### Themes generated through reflexive analysis

Data were organized into three main themes reflecting psychologists’ experiences with Muslim clients. These themes are, (1) The community context, (2) The Muslim client, and (3) The culturally informed psychologist.

### The community context

The first point raised by participants was the importance of understanding the context of the Muslim client. The context includes the cultural and religious background of Muslims as well as the historical context of South Africa. Participants noted that the cultural background, in conjunction with the historical context, informs the resulting mental health discourses in the Muslim community as described by their clients.

### Culture and mental health

Muslim participants noted that Islam values reflexivity and to their knowledge, Prophet Muhammad (pbuh)^3^ reflected and meditated daily. However, participants shared that working in the Muslim community demonstrates the opposite, “you know, life is a lot less reflective (P1).” Participants stated that clients reported on cultural norms of patience and enduring hardship. They observed that clients tend to understand and respond to distress from a religious standpoint that sometimes reflects interpretations that participants viewed as inconsistent with their own understanding of Islamic teachings, “I’m just thinking about the mom that committed suicide. Her dad was the GP, but the crap advice that she got from her gynae[chologist] and paed[iatrician], about you know, *sabr* (patience) and you must accept and whatever (P4).” The concept of *sabr* is used here to encourage enduring with patience, however, as explained by Islamic scholars, *sabr* is multi-layered and constitutes exercising restraint with God’s decree, but continuing action to address the situation and trusting the outcome (Arif and Sarwar, 2025).

### History and mental health

Participants stated that Muslims in South Africa form part of previously marginalized racial minorities. During Apartheid^4^, psychology was utilized as an instrument of social engineering, perpetuated racist ideologies and reinforced existing power structures (Yen and Vaccarino, 2018). Consequently, psychology, shaped by colonial histories, remains a discipline viewed with skepticism by many South Africans (Clay, 2017), as a population who endured the discriminatory laws of Apartheid South Africa (Pellicer and Ranchhod, 2022). These early experiences of Muslim families have shaped the current context:

There’s also a lot of generational trauma. I think that’s also the context of being Muslim and a person of color in South Africa…there’s a lot around that kind of socioeconomic circumstances, just economic deprivation, immigration…earlier on in families. (P9).

### Discourses on mental health

Participants described negative statements on mental health as shared by their Muslim clients, which often made treatment difficult. These statements can be traced to clients’ family interactions:

For most of them[clients], mental illness was not spoken about…within the family. For most of them, emotions certainly wasn’t a point of conversation in the family. Very little inquiries from caregivers about how they are actually feeling or how they are doing. (P15).

However, participants noticed a shift in how mental health is perceived in the Muslim community observed by working with Muslim clients over a number of years, “So, I think there has been more of a shift and a change around, people being aware of mental illness, the impact of it, and the need for professional treatment and help and support (P7)”. They attribute these shifts to Muslims entering professional spaces and being exposed to MH education thereby building MH literacy. Furthermore, they report suicides in the community and Covid-19 as contributing to the shift as it highlighted various MH needs and prompted Muslims to seek professional mental healthcare. Participants report an increase in the number of Muslims seeking psychotherapy as P14 stated:

I can definitely see a shift happening over the last couple of years especially. So, it seems almost like since COVID hit, people have been more open to it. I’m also seeing a lot older people, in my practice who actually really wants therapy.

Participants also commented on the difference in perceptions of MH and treatment among different age groups in the Muslim community. They report a distinct difference between older versus younger Muslims on how MHC are perceived and whether formal treatment is sought. P3 stated:

In the younger generation, I see a lot more seeking out for help, and actually valuing the importance of good mental health and emotional well-being and seeing it as Islamically, this is what they also need to do in order to get help. I see both rather, but as I’m saying in different population groups and also in age groups, there are different perceptions and views.

### The Muslim client

Two sub-themes are presented which demonstrate the experience of working with Muslim clients for psychological treatment. The first refers to how participants’ clients understood, perceived, and experienced MHC, and the second highlights the personal experiences of psychologists with their Muslim clients.

### Muslim clients’ explanatory models of mental health conditions

Participants reported several explanatory models for MHC according to their Muslim clients. Lack of religiosity and supernatural causes were the most significant. Lack of religiosity refers to a lack of or decrease in faith and spirituality facilitated by a perceived lack of religious practice.

Some of them would speak about, their *imaan* (faith) is not strong enough, at least that’s what they were told. And they should follow advice they might have gotten from others, which I think pertains a lot to actually just doing certain religious practices, you know, like increasing *solaah* (prayer) and those types of things. (P14).

Supernatural explanations were additionally perceived by clients as causes for MHC and included experiences of “black magic”^5^, “possession” by evil spirits, and “evil eye”^6^:

I have come across instances where they do kind of wonder or seek the explanation of where this *jinn* (evil spirit) or jealousy from other people (evil eye), that other people are sending all of these bad prayers and *vuil werk*^7^ (dirty work), and therefore one person in the family suffering or therefore, I’m suffering or I’m in distress” (P12).

Participants also observed that madness was often perceived as an explanation of psychological distress by their clients. Madness was typically associated with individuals who presumably experienced hallucinations and delusions and were subsequently feared due to perceived unpredictability and violent behavior. While this explanation may not be specific to the Muslim community, it underscores a misconception about MHC:

Growing up, it was the crazy people who have mental illness. Those who are mad and crazy and talk to themselves and hurt people and just the crazy people…and if you go to the psychologist then they’ll put you in a facility and you are gonna have to be strapped. (P11).

Lastly, weakness was also an explanation referring to an individual’s inability to manage their daily functioning. Weakness here may be referring to psychological weakness, which is defined as impaired coping and emotion dysregulation (Scandurra et al., 2024), the opposite of enduring through hardship, like their forefathers did during Apartheid. Participants stated that while many clients have revised their explanations of MHC, the explanations reported still exist within the Muslim community at large and influence health-seeking behavior.

There’s that understanding that it can be avoided, and you are just weak. You need to suck it up and you need to deal with it. When we grew up, we did not have those things. We just pushed through and why cannot you do it now? People are lazy. People do not know how to be tough now. All this bunch of weaklings over here because now mental health is a thing. (P11).

Participants reported three prominent emotions associated with their clients’ mental health experiences. These emotions were fear, guilt, and shame. Muslim clients often feared rejection from their family, the community, and society due to perceived inherent abnormalities. This fear often influenced their commitment to therapy and determined whether they remained adherent to treatment: “And it leads to embarrassment, fear, rejection in some cases, within the family and the community, and also like, if I think about vulnerable individuals not necessarily getting the support and the help that they do need” (P3). Participants reflected that guilt was also associated with MHC and clients feared bringing shame to the family through their diagnoses, as MHC were considered to be a sign of poor religiosity. Many of their clients also experienced shame because they perceived themselves as not being good enough Muslims. Ultimately, the guilt and shame were centered on the MH diagnosis, because it was experienced as something unacceptable and meant they did not measure up to the perceived *ideal* Muslim.

I had clients who were OCD, who were close to it, who were just kind of wracked with guilt. And so, they would just always, kind of feeling like they were not what they were supposed to be, and they were not thinking in the right way. (P1).

Beliefs and attitudes form part of EMs according to Kleinman. Participants perceived that clients grew up in a community that believes they are immune to MHC, and therefore professional help is not sought: “It’s like mental health is still out there for the sort of ‘white people thing’, versus like saying all of us are susceptible to mental health conditions” (P4). This is not an unusual stance considering the lived realities and psychological needs of the marginalized Black communities were ignored during Apartheid (Clay, 2017). Psychological support is thus viewed as only accessible and utilized by ‘white’ South Africans. Furthermore, issues are often externalized, resulting in a lack of accountability:

Not really knowing where the problem comes from, so maybe rather thinking about, looking for external, like more obvious problems. It’s like, OK, if I just change my job, if I just got divorced or whatever, versus being able to look at the depth, underneath whatever it is that’s the problem. (P5).

Participants also shared that Muslim clients highlighted how MHC are perceived to be unacceptable for a Muslim, and so psychological treatment is considered unacceptable as a treatment option. Muslim clients who do seek psychological treatment often attend in secret: “So, they very secretly come (P6),” and diagnoses and treatment are not disclosed to the family:

Some of them are older adults. They’re married, have their own families, but they have not shared, especially their family of origin, that they are in therapy. And then we have ducking and diving…because the parents do not know, because they are afraid of the backlash. (P11).

Participants emphasized that according to their clients, MHC are still highly stigmatized in the Muslim community:

And that I’m seeing a psychologist itself will also be an issue, and that itself is stigmatizing, so, I witnessed rather that clients needed to suffer in silence and like OK, so I’m seeing a counselor, but I’m not very open about this. (P3).

Participants reported that these explanations, beliefs and attitudes inform health-seeking behavior. According to their clients, treatment thus often included *spiritual cleansing*^8^ by religious leaders: “And they may need to see rather a spiritual leader and an *imam* (cleric) or some other person rather than actually a psychologist, because the issue is not a psychological one, but rather religious or a spiritual issue” (P4). In addition, participant’s clients focused on improving religiosity by increasing faith through an increase of religious practices, such as prayer, recitations, reading the *Qur’an*^9^, and other non-obligatory religious practices. P8 stated:

And then inevitably, their first decision when they do go through the difficult times is, let me start doing everything, praying five times a day, doing the extras, going to mosque, whatever it is that they kind of need to do, they usually go to that first. Because they feel that when times are tough, you return to your creator, and that’s where you will find solace.

Focusing on religiosity as a treatment sometimes contributed to health risks:

I’m talking about, to the point of suicide ideation and self-harm. Her parents were aware of it, but did not take action because her dad was just like, oh no, you must go make *solaah*^10^ (pray), you know? So, that’s the rhetoric I hear often. just go pray it away, just go make *solaah* (pray) and you’ll be fine. (P7).

However, participants noted a shift, with clients starting to recognize the importance of professional treatment for MHC, motivated by principles of Islam. They further noted that those who sought formal healthcare valued the therapeutic space:

But then in a lot more of the younger generation, I see a lot more seeking out for help, and actually valuing the importance of good mental health and emotional well-being and seeing it as actually, Islamically, that this is what they also need to do in order to get help. (P3).

One participant observed that many Muslims still preferred to engage in self-help practice rather than seeking professional treatment: “I think that many people are also keen on rather doing self-help, books or coaches, than actually seeing a counselor or a psychologist (P3).”

### Treatment experiences with Muslim clients

Participants observed that for their clients, Islam is typically used as the key framework for life, and informs the majority of their decisions, even if they do not identify as practicing Muslims:

I find that with my Muslim clients, they quite quickly talk about the effect of religion on their lives and the context of Islam, particularly Islam in Cape Town. So that’s one of the things that really comes up, the religion is quite dominant, or at least prevalent in what they bring to therapy. (P1).

Secondly, participants stated that in their view Islam often serves as a protective factor for clients: “I think that religion often forms a big anchor in therapy, and often it’s a protective factor. And I think not understanding that really puts you at the deficit in treating Muslim patients (P6).” Furthermore, they believe faith aids clients’ resilience because difficulties are interpreted through a religious framework:

In terms of faith and reliance on a higher power, just kind of knowing that they are not walking through this alone, that there is a God, there is *Allah*^11^ and kind of even though you know they open up and they speak about the difficulties, there’s always that reliance and trust and faith in *Allah*. (P12).

Finally, Islam *can* present challenges in treatment, as interventions perceived to conflict with religious beliefs may be rejected. P6 stated:

I find that to be the biggest challenge, I suppose, with the people that I’ve seen, that they are devout in their faith. And if they think that any decision would jeopardize their faith, they would rather just endure whatever hardship they have to because for them, the balance is, I’ll choose my faith over whatever it’s gonna cost me.

Participants emphasized that subjective interpretations of Islam are often used to maintain the status quo with clients. These factors hinder accountability and deeper engagement with psychological treatment, which is necessary for progress:

But religion in general was definitely a barrier because it felt like, to some or other degree, you could rather kind of turn to religion, not only for encouragement or solace or support or whatever, but also for an explanation. And it’s an explanation that helps you to defend against the realities of what’s actually in you, and what you could take responsibility for, and what you could actually do something about. (P5).

Participants shared varying experiences with clients. Many perceived that clients tended to prefer Muslim psychologists: “Muslim people generally seek out Muslim therapists, I think for depth of understanding of religious practices and *Shariah*^12^ law (P11).” Participants believed this seeking out was to ensure their religion and culture would be understood: “Often clients will phone in and request a psychologist (A) that is of color and (B) that is Muslim (P2).” Participants acknowledged Muslim clients who were able to engage well with psychological treatment: “But it’s almost like they can set it aside and still engage in a therapeutic process (P14).” However, participants additionally acknowledged experiencing resistance from numerous clients, due to ambivalence about seeking formal healthcare instead of seeking help from their God: “And also similarly a conflict with them, rather an ambivalence and a resistance to seeking help because not being sure whether they are doing the right thing (P3).” Participants also reflected that some clients perceived therapy as a threatening space because it was unfamiliar: “it just feels like really threatening for a lot of people to now engage in a space that’s open and not defined by the parameters of either religion or cultural family dynamics (P9).” This perception often resulted in premature treatment termination:

So, what happens is like little lightbulbs go on, sometimes that’s scary for them and then they just do not come back. They kinda run because they are like oh OK, it wasn’t just that I did not make *solaah* (pray) or this or that or whatever. (P4).

Participants reported that seeking formal treatment was often a new experience for clients which contributed to the struggle with navigating formal treatment, “I’ve never been to therapy before, I’ve no idea what to expect (P5).” They further highlighted a difference in how older and younger clients engaged, with older clients prone to struggling in therapy:

And I’d find the older clients like in their forties, and much older than that, they put less thought into what actually influences their mental health. So, they come with an issue and they say this is how I feel, but then I’m left to make sense of OK, so where does this possibility stem from, what kind of factors have influenced where you are right now. (P15).

Lastly, participants emphasized Muslim clients’ conflict where religion and mental health intersect.

There’s like a conflict sometimes also with, I’m struggling with this, but I need to hide it because the understanding of what should make me feel better is religion. I do not think that’s all my clients, but that’s sometimes quite prevalent. (P1).

Participants also reported external challenges that influenced client treatment. Muslim families still largely refuse to acknowledge the value of psychological treatment: “But then people would also still speak about, maybe like a parent or whatever, not really believing in therapy or being against therapy even (P14).” Sometimes clients’ families and community expectations are at the root of difficulties, “I think that’s also challenging and that’s obviously another whole conversation which is about the parental expectations and the religious expectations on children and adolescents and how that affects the younger generations’ mental health also (P9).”

Lastly, culture is frequently confused with religion and some practices are perceived as compulsory to increase devoutness. This misperception hinders treatment and contributes to maintaining psychological distress:

I think if I had a deeper understanding of Islam, I might be able to identify what is actually Islamic and what is cultural, easier. But I definitely think, especially within the Western Cape, because there’s such a big Cape Malay culture, which has a lot of Islamic influences, but it is not necessarily in its totality Islamic, that I think there is a deep enmeshment when it comes to the two and we just assume that the one means the other. And I think people follow those kind of cultural rules because of their devotion to their faith and they think that this is what’s gonna make them more devout within their faith. (P6).

### The culturally sensitive psychologist

Participants emphasized the importance of developing cultural competency for clinical practice. Most participants working with Muslim clients were Muslim themselves and reflected drawing on their own knowledge and experiences as a resource. Participants also described strategies they used and provided further recommendations for developing cultural sensitivity.

### Developing cultural competence in psychological practice

Participants acknowledged that even rudimentary knowledge of Islam was beneficial: “You know, also just certain terminology or experiences or events that I do not have to necessarily go into explaining all the details (P3).” However, they engaged with religion in therapy only when it was appropriate to do so:

I suppose because [I] identify as Muslim, I think there’s always gonna be that part I would bring, thinking about OK how am I supporting and helping this Muslim person. I think I tried to bring it into the treatment program if I think it’s appropriate to the case and if it’s gonna be helpful for the patient. (P8).

Moreover, participants reported no discernible difference between their treatment approaches and those of their non-Muslim colleagues, or with non-Muslim clients. However, understanding the context and religious nuances facilitated rapport-building and enhanced open communication:

It’s not necessarily that we would work from a very Islamic psychology perspective. I mean, I’m not even trained in that. So, we do not do that. We would work more traditionally in terms of the traditional models of psychotherapy. I think just having that overall knowledge and context and idea of the culture and religion, I think helps. (P9).

Some participants posited that clients felt more comfortable bringing religion into the treatment space when they perceived a shared religious affiliation:

I find that now that I’m wearing a scarf compared to when I wasn’t wearing a scarf and I was doing the same job I’m doing now, I find that patients bring in religion a lot easier than when I wasn’t in *hijab* (headscarf). (P10).

On developing religious competency, participants discussed engaging with Muslim colleagues and supervisors as a resource:

I was very lucky to have lots of contact with fellow Muslim clinicians. And I think since the beginning of my training that was there. So, in discussing patients or things that came up or things that felt out of sorts, I think I always had that point of access to go and check and double-check myself and gain an understanding that I might be missing. (P6).

Several participants described consulting with Muslim clerics, particularly in understanding the Islamic worldview: “It helps because I’ve got a *sheikh* (cleric) in the office with me (P2).” Lastly, several participants noted educating themselves on Islam either through accessing online courses and lectures, or in-person talks by Islamic scholars. They noted that this kind of information is more easily accessible now than earlier in their careers and is helpful when working with Muslim clients to enhance treatment:

I feel like going out and trying to get more accurate information that are based on *hadiths*^13^ or those kind of things…I feel that the more accurate information I’ve been able to learn, that the better it’s actually made me able to help my patients who do have a stronger religious aspect to their being…I’m able to help them better. (P10).

### Approaches to culturally sensitive care

Participants described a curious but cautious approach, emphasizing a conscious effort to avoid assumptions and judgments, especially since many participants shared a religious identity with their clients:

I think that, it’s been really important for me in my practice, you know, a sense of openness to being curious about what their experience is rather than making an assumption that OK, this is my experience as a Muslim in Cape Town. (P7).

Participants reflected on the importance of maintaining professional boundaries, but leaning into their relatability which aided rapport building: “So just that importance of rapport building and not rushing through it, but also to be very careful not to collude with avoiding therapy (P5).” Participants also found that maintaining religious sensitivity from the onset helped clients settle into the ‘formal’ space: “I feel that some Muslim clients who come in, in order for it to make sense to them, they need you to work from a very spiritual place (P13).”

However, they cautioned against a one-size-fits-all approach: “and then you have other clients who do not want that at all. So, I definitely feel I have to adapt based on what their needs are (P13).” Providing psychoeducation was identified as a significant part of treatment, with participants emphasizing the need to reframe and normalize MH experiences.

So a lot of psychoeducation around these kind of things and how they can be useful and how they are also in line with you being a Muslim person and looking after yourself, because that’s also something that you need to do. So I try to reframe that. (P11). In addition, participants reiterated the importance of acknowledging supernatural explanations, such as spirit possession, black magic and evil eye, as part of Muslims’ belief system and working with clients to understand how to navigate the intersection of Islam and mental health.

I think again, this is when you must work with a good psychiatrist for these kind of things, because you need to be able to say like I hear you, but let us rule out all the medical stuff because these things do, Islamically they exist, but we also know in mental health that it could be psychiatric and so we have to have it checked out. (P4).

### Recommendations for culturally sensitive care

Participants shared the view that an essential first step to developing culturally sensitive care is to educate themselves as practitioners. P1 stated: “And I feel like we need to educate ourselves more,” and “most importantly is that it’s the clients themselves that educate you” (P2).

Participants also shared the opinion that reducing stigma would help with bridging the gap between mental health practitioners and the community. They believed religious leaders were necessary for this step to start normalizing mental health needs in the Muslim community:

I just feel that if it was less of a taboo subject to talk about, it would go a long way for people to get earlier intervention, than wait till things become as severe, where it’s a lot harder to undo some of the damages that have been done or unlearn some of the things that have not been helpful. (P10).

Lastly, participants emphasized collaborative efforts for the benefit of the Muslim community: “It’s like we need the same collaboration between mental health professionals and Muslim clerics (P7).” They additionally recommended building a support network when working with Muslim clients:

I think the other issue is we often think of culture as being a static thing, and that’s rubbish. Culture is innately dynamic. And so, you need to stay afresh with the *dynamicness*. Now, how do you do that? Through having your support systems in place. (P2).

Participants proposed several recommendations for developing culturally sensitive approaches in psychological practice.

## Discussion

This study aimed to understand the experiences of psychologists providing psychotherapeutic services to Muslim clients and explore psychologists’ accounts of explanatory models of MHC as expressed by their Muslim clients.

This is the first contemporary study located in the Western Cape region documenting psychologists’ engagement with Muslim explanatory models in a psychotherapeutic space in post-Apartheid South Africa. This study also extends Kleinman’s theory by demonstrating that explanatory models include relationships to institutions and collective histories, not merely beliefs about illness causation. Furthermore, while previous South African studies have documented religious and supernatural explanatory models among Muslims, the present study demonstrates that psychologists encounter these explanatory models as deeply embedded within broader histories of racialization, migration, intergenerational trauma, and institutional mistrust. These findings suggest that explanatory models may reflect not only beliefs about illness but also collective historical experiences and relationships with systems of care.

Participants reflected on the historical context of South African Muslims, noting that the legacy of Apartheid, including experiences of deprivation, discrimination, oppression, and exclusion from basic services, continues to shape perceptions of psychology, psychologists, and mental health (Mayet and Phiri-Sithole, 2025). Participants suggested that psychology, and by extension psychologists, are often viewed with skepticism by members of historically racialized and marginalized communities. Consequently, Apartheid legacies, migration histories, and intergenerational trauma appear to inform community understandings of MHC and influence help-seeking pathways. These findings underscore the need to move beyond simplistic cultural categories (Kleinman, 2013) and recognize the complexity and diversity of Muslim communities, whose experiences are shaped by intersecting historical, social, and religious influences (Syed and Pio, 2018). Moral experiences, one of Kleinman’s constructs (REF), was repeatedly brought up in therapy by Muslim clients. Lack of religiosity and supernatural explanations were most prominent and therefore treatment was sought from religious leaders or attempts were made to improve religiosity through increased religious practices. Illness narratives described by psychologists included beliefs that Muslims were immune to MHC; MHC were unacceptable and stigmatized, and Muslims diagnosed with MHC experienced fear, guilt, and shame. This finding supports similar findings by Tanhan and Young (2022); Verwey (2024), highlighting religious interpretations of MHC which shape attitudes toward mental health and influence willingness to seek professional help. These feelings are often exacerbated by religious and cultural beliefs, leading individuals to avoid discussing their mental health issues openly and navigating their experiences in isolation as indicated by psychologists reporting that their clients often remained secretive about seeking formal mental healthcare. The findings suggest that explanatory models among Muslim clients are not limited to causal beliefs about illness. Rather, the models include collective historical narratives, moral identities and institutional relationships.

It was clear from participants that Islam was salient for their Muslim clients, regardless of their perceived religiosity, and was often dominant in treatment discussions. Participants emphasized that Muslims’ belief in and reliance on a higher power often served as a protective factor, providing them with psychological resilience and aiding them in coping with the challenges they experienced in their daily lives. However, Islam played a paradoxical role for participants’ Muslim clients. Islam provided a source of resilience, helped with meaning making of experiences related to MHC, provided hope and mental health experiences were perceived as opportunities to gain closeness to God. At the same time, Islam was used to justify avoidance of help-seeking, it was often used as the sole explanation for distress, and it was used to maintain status quo. When the latter was prominent, MHC was perceived as a threat to Muslim identity, resulting in feelings of guilt and shame. Psychologists were thus constantly negotiating between explanatory models in the therapeutic space—between biomedical, Islamic and client understandings.

Participants also described what they perceived as a frequent conflation of cultural practices and Islamic teachings among some Muslim clients, a phenomenon that is particularly salient within broader debates on religion, culture, and identity (Scheer, 2022; D’Urso and Bonilla, 2023; Becker et al., 2023; Sriram and Westfall, 2025). Importantly, participants did not identify religion itself as the primary barrier to help-seeking. Rather, they suggested that certain cultural expectations and practices were accorded religious legitimacy and understood as obligatory, which at times discouraged engagement with formal mental healthcare. Participants viewed this as a significant challenge within therapy, consistent with previous research highlighting the complex intersections between religion, culture, and mental health help-seeking (Ghuloum et al., 2024; Sikrweqe et al., 2024; Alqasir, 2024). The current study demonstrated this complexity where Islam featured prominently, but so did Muslims’ generational traumas.

Lastly, participants provided several pathways for developing cultural humility learnt through their own experiences working with Muslim clients. They highlighted the need for HCPs to educate themselves on religion and culture, and emphasized clients as a valuable educating resource. Kleinman (2013) highlighted the importance of empathy and active listening in providing culturally sensitive care. By genuinely listening to clients, HCPs can gain a better understanding of client values, beliefs, and experiences. This approach helps in building trust and providing care that is respectful and responsive to individual needs. Further resources noted for developing cultural sensitivity included Islamic material sourced through online resources by reputable scholars, as well as Muslim colleagues. This strategy echoes recent studies that recommended consultation with religious leaders, often the first point of contact for Muslims when in distress, and Muslim therapists as they are knowledgeable about Islam and the communities they come from (Hodge et al., 2023).

Taken together, the findings suggest that explanatory models are not isolated beliefs about the causes of MHC. Rather, psychologists described explanatory models as situated within interconnected historical, sociocultural, religious, and institutional contexts. Figure 1 presents a conceptual framework illustrating how broader structural influences, including Apartheid legacies, migration histories, racialization, and intergenerational trauma, interact with family expectations, religious worldviews, community norms, and stigma to shape explanatory models of distress. These explanatory models subsequently influence emotional experiences, help-seeking pathways, and engagement within the therapeutic encounter.

**Figure 1:**
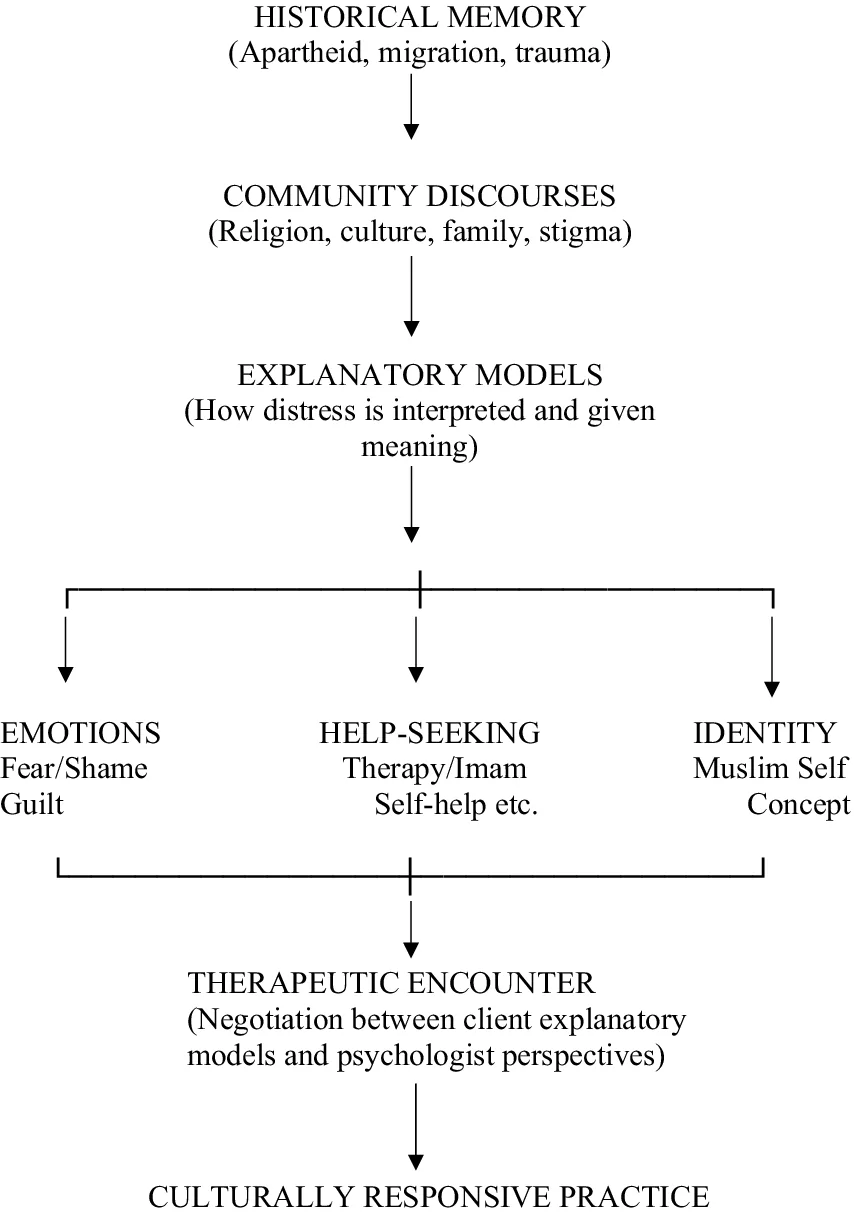
Figure depicting conceptual framework.

This framework emphasizes that explanatory models function not merely as causal attributions but as broader systems of meaning through which distress is interpreted, negotiated, and managed. Psychologists described therapeutic work as involving ongoing negotiation between clients’ explanatory models, community narratives, religious understandings, and professional psychological perspectives.

Through a social constructionist lens, the findings demonstrate how shared meanings, and cultural norms continue to inform actions, including with mental health experiences despite the negative help-seeking consequences. These cultural norms exacerbated challenges for Muslim clients living with MHC. Although MHC are often misconceptualized in Muslim communities (Shah et al., 2014), like previous studies (Hodge and Nadir, 2008; Khan, 2014; Shah et al., 2014), this current study found that Muslim clients responded well to openness and respect for religion and acknowledging their faith as a positive source of strength.

It is noteworthy that most participants in this study were Muslim. While participants noted no difference between their treatment approaches and their non-Muslim colleagues, the majority stated that in their experience, Muslim clients sought out Muslim psychologists.

Participants stated that Muslim clients appeared to value the psychologist’s understanding of their context, such as understanding Islamic practices and religious rhetoric, cultural nuances, and also historical context. As one participant noted, Muslim clients seek practitioners who are either Muslim or persons of color. These findings are supported by other studies, for example, Hodge et al. (2023); Hassan et al. (2025); Islam and Chadwick (2025) and Khan (2014), where Muslim psychologists were perceived as knowledgeable about Muslim communities. However, in this instance, Muslim clients were comfortable with psychologists of color as well, because they believed psychologists of color would also understand the sociopolitical and historical context.

These findings represent one stakeholder perspective within a broader multi-stakeholder PhD study exploring explanatory models among Muslims in the Western Cape. Future publications draw on interviews with Muslim community members, service users, and Islamic scholars, allowing comparison across explanatory frameworks.

## Conclusion

This study explored the experiences of psychologists providing psychotherapeutic services to Muslim clients as well as psychologists’ accounts of explanatory models of MHC reported by Muslim clients. Psychologists described navigating a complex environment where religion, culture, history, and intergenerational experiences converge to influence perceptions of MHC and the process of seeking help. Explanatory models shared by Muslim clients often encompassed a lack of religiosity, supernatural explanations, personal weaknesses, and the belief that MHC contradicts their Muslim identity, which frequently led to stigma, secrecy, fear, guilt, and delays in seeking help. Conversely, religion also served as a significant source of resilience, meaning, and coping strategies. Beyond documenting psychologists’ accounts of explanatory models expressed by Muslim clients, this study contributes to explanatory-model scholarship by demonstrating that understandings of MHC are embedded within collective histories, community discourses, and relationships with institutions. The findings suggest that culturally responsive psychological practice requires engagement not only with religious beliefs, but also with the historical and sociopolitical conditions that shape how distress is interpreted, experienced, and treated within Muslim communities in the Western Cape.

### Clinical implications

The findings support calls for healthcare professionals to develop culturally responsive practice through ongoing education, ethical reflection, and engagement with diverse knowledge systems. In the South African context, this includes recognizing religious and indigenous understandings of mental health as meaningful frameworks that shape experiences of distress, help-seeking, and recovery. Such recognition is particularly relevant to efforts to decolonize mental healthcare and move beyond approaches that privilege biomedical explanations over clients’ lived realities.

Participants’ accounts suggest that culturally responsive practice requires more than familiarity with Islamic beliefs and practices. Psychologists may benefit from developing their capacity to explore clients’ own explanatory models of distress while recognizing the diversity within Muslim communities and avoiding assumptions based solely on religious identity. This may include creating space for discussions about religiosity, supernatural explanations, spiritual struggles, and religious coping in ways that acknowledge their significance without pathologizing such beliefs or assuming authority over theological matters. A stance of curiosity and cultural humility may assist clinicians in understanding how clients’ make meaning of their experiences and how these meanings shape treatment engagement.

The findings also underscore the importance of understanding the broader historical and sociopolitical context within which explanations of MHC are formed. Awareness of racialization, intergenerational trauma, migration histories, socioeconomic marginalization, and historical distrust of institutions may assist psychologists in situating clients’ experiences within their broader context. Attention to these influences may strengthen therapeutic alliances and provide a richer understanding of ambivalence, resistance, or delayed engagement with care.

Participants further described the need to navigate situations in which religious and psychological understandings of distress coexist or appear to diverge. This includes recognizing faith as both a source of resilience and meaning-making and, at times, a contributor to guilt, shame, fear, or reluctance to seek help. Rather than viewing religious and biomedical explanations as mutually exclusive, it may be helpful to support clients in navigating multiple frameworks of understanding in ways that align with their values and needs.

Ongoing opportunities for reflexive supervision, consultation, and professional dialog may further support culturally responsive practice by helping psychologists critically reflect on assumptions, avoid stereotyping, and respond more effectively to complex cultural and religious presentations.

The findings also point to the potential value of collaboration between psychologists and Muslim religious leaders, who are often among the first sources of support accessed by individuals experiencing distress. Where such collaboration is appropriate, attention to informed consent, confidentiality, role clarification, referral pathways, and the management of differing clinical and religious perspectives may help facilitate effective working relationships. Thoughtful collaboration may contribute to earlier intervention, reduced stigma, and more integrated systems of support.

Overall, the findings suggest that culturally responsive care for Muslim clients requires attention not only to religious beliefs and practices, but also to the historical, social, cultural, and political contexts within which these beliefs acquire meaning. Greater attention to explanatory models, religious narratives, historical sources of mistrust, and interdisciplinary collaboration may contribute to more meaningful therapeutic engagement and responsive mental healthcare for Muslim communities.

### Limitations

Firstly, majority of the participants were Muslim. This self-selection may have resulted in an overrepresentation of culturally sensitive psychologists with privileged insider perspective, thereby obscuring challenges that may be experienced by psychologists less familiar with Islam. Secondly, Muslim psychologists are able to provide richer insights as they are more familiar with Islam and more attuned to religious nuances. However, this also means that a bias exists for faith-based EMs and integrating religious understandings. Participants thus already represent a culturally responsive sub-group. Thirdly, the study is based in one province of the country with a unique sociocultural and political history. Findings may not directly transfer to other Muslim communities in South Africa as Muslims are heterogeneous with differing sociocultural and political histories across the provinces.

Lastly, findings demonstrate participants’ interpretations of their clients’ experiences. These interpretations are informed by professional training, personal beliefs and clinical encounters. These insights are valuable but should be considered alongside other stakeholder insights. Although there was a wide recruitment process across multiple psychology networks, most of the psychologists who responded were Muslim, because they primarily work with Muslim clients.

## Data Availability

The original contributions presented in the study are included in the article/supplementary material, further inquiries can be directed to the corresponding author.

## Footnotes

1 A culturally embedded set of beliefs involving unseen metaphysical forces, such as jinn, spirit possession, the evil eye, black magic, or divine/interventionist agents, invoked to explain psychological distress or behavioral changes. These beliefs form part of a cosmology where the boundaries between mental, spiritual, and physical health are porous and interpreted through religious, cultural, and explanatory models of illness (Lim et al., 2018).

2 Independent practitioners are registered with the health professions council of South Africa (HPCSA) to practice in their relevant health profession in South Africa. This registration indicates successful completion of the academic qualification and the necessary board exams, and therefore valid credentials. The training period to reach this stage is 7 years in total, where the last 3 years includes substantial practical hours.

3 Peace be upon him. A phrase of reverence used by Muslims when mentioning the Prophet’s name.

4 From 1948 until the early 1990s, the South African government implemented an institutionalized system of racial segregation and discrimination known as apartheid. The “white” minority’s political and economic dominance over non-white communities was intended to be maintained under the system (UNESCO, 1967).

5 Black magic refers to the practice of using supernatural or occult powers to harm, manipulate or control others (Khalifa et al., 2011).

6 Evil eye refers to a cultural belief of someone being harmed or afflicted by the envy, malice, or negative energies of others, intentionally or unintentionally, through a gaze, glance or thought (Ayshi et al., 2015).

7 A colloquial term used to reference engaging or collaborating with the supernatural world in a negative way as it is intended to harm someone.

8 A Qur’an-based, prophetically endorsed treatment or healing method that employs recitation, prayer, and supplication to address physical, emotional, and spiritual distress within Islam (Al Ghiffary et al., 2021; Mohd Hamdan et al., 2025).

9 Muslims’ holy book of scriptures.

10 Solaah refers to the act of prayer performed by Muslim people daily (Sarkingobir et al., 2022).

11 The Arabic term for God in Islam.

12 Divine law in Islam, according to the Qur’an and prophetic tradition.

13 Arabic term for the collection of traditions in Islam comprising of sayings, actions and approvals of Prophet Muhammad (pbuh), serving as one of the primary sources alongside the Qur’an for Islamic law, ethics and theology.
